# Efficacy of Diltiazem for the Control of Blood Pressure in Puerperal Patients with Severe Preeclampsia: A Randomized, Single-Blind, Controlled Trial

**DOI:** 10.1155/2020/5347918

**Published:** 2020-07-23

**Authors:** Gilberto Arias-Hernández, Cruz Vargas-De-León, Claudia C Calzada-Mendoza, María Esther Ocharan-Hernández

**Affiliations:** ^1^Hospital De La Mujer, Prolongación Salvador Díaz Mirón 374, Colonia Santo Tomas, Delegación Miguel Hidalgo, C. P. 11340, México D. F., Mexico; ^2^Facultad De Matemáticas, Universidad Autónoma De Guerrero, Chilpancingo, Av. Lázaro Cárdenas S/N, Cd. Universitaria, 39087 Chilpancingo, Guerrero, Mexico; ^3^Instituto Politécnico Nacional Escuela Superior De Medicina, Plan De San Luis Y Díaz Mirón SN, Col. Casco De Santo Tomás, Delegación Miguel Hidalgo, C. P. 11340, México D. F, Mexico

## Abstract

**Background:**

Postpartum preeclampsia is a serious disease related to high blood pressure that occurs commonly within the first six days after delivery.

**Objective:**

To evaluate if diltiazem improves blood pressure parameters in early puerperium patients with severe preeclampsia. *Methodology*. A randomized, single-blind longitudinal clinical trial of 42 puerperal patients with severe preeclampsia was carried out. Patients were randomized into two groups: the experimental group (*n* = 21) received diltiazem (60 mg) and the control group (*n* = 21) received nifedipine (10 mg). Both drugs were orally administered every 8 hours. Systolic, diastolic, and mean blood pressures as well as the heart rate were recorded and analyzed (two-way repeated measures ANOVA) at baseline and after 6, 12, 18, 24, 30, 36, 42, and 48 hours. Primary outcome measures were all the aforementioned blood pressure parameters. Secondary outcome measures included the number of hypertension and hypotension episodes along with the length of stay in the intensive care unit.

**Results:**

No statistical differences were found between groups (diltiazem vs. nifedipine) regarding basal blood pressure parameters. Interarm differences in blood pressure (systolic, diastolic, and mean) and heart rate were statistically significant between treatment groups from 6 to 48 hours. Patients in the diltiazem group had lower blood pressure levels than patients in the nifedipine group. Significantly, patients who received diltiazem had fewer hypertension and hypotension episodes and stayed fewer days in the intensive care unit than those treated with nifedipine.

**Conclusions:**

Diltiazem controlled arterial hypertension in a more effective and uniform manner in patients under study than nifedipine. Patients treated with diltiazem had fewer collateral effects and spent less time in the hospital. This trial is registered with NCT04222855.

## 1. Introduction

Both preeclampsia and eclampsia are important health problems, being important causes of maternal death worldwide [1–3]. Although these two pregnancy-related disorders have been widely studied, their cause remains unknown. Pathophysiological mechanisms involve the reduction of maternal placental blood flow as of week 20 of gestation, with an increase in peripheral vascular resistance that predominantly takes place in the kidney, liver, and brain [4–9]. It has been reported that placenta plays a key role in the pathogenesis of preeclampsia, and it was assumed that, after delivery, its influence in the maternal cardiovascular system would be over. However, this does not occur, and it remains during early puerperium (from 24 hours, mostly, to 6 days after delivery) [4, 9].

Severe preeclampsia is defined as “artery pressure greater than or equal to 160/110 mmHg in at least two determinations with less than two hours of difference. Patients may present headache, acute lung edema, blurred vision, phosphenes, right flank pain, vomiting, liver hypersensitivity, HELLP syndrome, thrombocytopenia, and elevated liver enzymes (ALT or AST)” [10].

Antihypertensive drugs in pregnancy, including oral nifedipine (10 mg), methyldope (250 mg), labetalol (100 mg i.v.), and furosemide (20 mg OD), are used during postpartum too [11–14].

Arterial hypertension that accompanies severe preeclampsia is caused by an increase in peripheral vascular resistance. For this reason, calcium antagonists are ideal candidates for antihypertensive treatment in these patients. [14–17].

Nifedipine has been used as the drug of choice for the treatment of hypertension during puerperium for more than 25 years [14, 17]. However, there are several studies that report the presence of serious adverse effects with the use of this drug, such as cerebral ischemia, myocardial ischemia, or tachycardia, as well as episodes of hyper- and hypotension [18, 19]. Consequently, it is necessary to find treatment alternatives for these cases.

Diltiazem is an alternative calcium antagonist that is 1000 times less potent than nifedipine, which has the property of producing a selective vasodilatation of arteries beds [20–25]. Thus, it has little effect on the venous return and cardiac function, allowing for better cellular perfusion than that found with nifedipine [21, 22]. Many studies report that diltiazem has a wide safety margin and few side effects due to its pharmacological properties [21, 23]. On this basis, a clinical trial was carried out to test the efficacy of diltiazem in treatment of hypertension of early puerperium patients with severe preeclampsia.

## 2. Materials and Methods

### 2.1. Study Population

A randomized, single-blind, longitudinal clinical study was carried out with patients in early puerperium (defined as the first 24 hours after delivery) [26, 27] without hypertension antecedents during pregnancy, but were diagnosed with severe preeclampsia, since they presented two of the criteria (systolic blood pressure ≥ 160 and diastolic blood pressure of 110 mmHg) indicated in the Clinical Practice Guide of the Mexican Institute of Social Security [10], in the intensive care unit of the “Hospital de la Mujer” from Mexico's Secretariat of Health (SSA). Inclusion criteria: for all patients, the following parameters were within the normal range: hematic biometry, urine and glucose tests, aspartate aminotransferase (AST), alanine aminotransferase (ALT), serum creatinine, and the blood coagulation test including prothrombin time and activated partial thromboplastin time. Electrocardiogram and chest radiography showed no pathological findings. Patients with edema and headache were also included. Exclusion criteria: when the patient falls outside of the aforementioned parameters or subjects with unstable medical conditions.

All patients signed an informed consent prior to their participation on the study. The study is registered in ClinicalTrials.gov with identifier number NCT04222855.

### 2.2. Randomization and Blinding

A researcher not involved in the study performed the randomization by using a random-numbers table and central allocation. Treating study physicians, nurses, and study investigators were not blinded to group assignment, whereas study participants were blinded to treatment allocation. Patients were randomly allocated to one of the two treatment groups: the group one was administered 60 mg of diltiazem (tables), while the group two (the control) was administered 10 mg of nifedipine (capsule). Both drugs were given orally every 8 hours.

### 2.3. Outcomes

Primary outcome measures were blood pressure parameters. Systolic, diastolic, and mean blood pressures and heart rate were recorded at baseline and after 6, 12, 18, 24, 30, 36, 42, and 48 hours. Secondary outcome measures included the number of hypertension and hypotension episodes and ICU (intensive care unit) length of stay.

### 2.4. Data Collection and Patient Evaluation

A Foley catheter was placed to measure urinary excretion. Also, to continuously measure the blood pressure of all patients, a catheter was placed in the radial artery and recorded with a monitor (DASH 4000 Dinamar, US). It provided readings of systolic, diastolic, and mean blood pressure. Readings were taken during the 48-hour observation period in order to ensure that blood pressure was maintained between 120/70 and 160/110 mmHg, and to either determine hypotension (defined as the pressure level at which clinical alterations occur) or hypertension events, according to a Cochrane systematic review [28]. For patients outside of this range, rescue therapy was applied; in case of hypertensive crisis, 10 mg of hydralazine was administered intravenously three times (every 20 min); while, in case of hypotension crisis, 200 ml of crystalloids solution was administered until the patient stabilized. After successfully controlling blood pressure, patients continued assigned treatment.

An electrocardiogram was performed to determine the heart rate. A solution was administered at the rate of 100 ml/hr to liquid handling, and this flow was adjusted according to central venous pressure measurements and laboratory tests results.

### 2.5. Sample Size

Sample size was determined by using two-independent samples *t*-test formula. For calculation, we used systolic pressure values derived from a pilot study of 10 subjects for each group. The common standard deviation of delta values (pressure differences between baseline and after 72-hour) is 19 mmHg, difference between delta values of treatments is 16.5 mmHg, and power level of 0.80, resulted in 21 patients per group.

### 2.6. Statistical Analysis

Descriptive statistics (mean and standard deviation for quantitative variables and absolute and relative frequency for qualitative variables) were used to summarize the clinical and biochemical features of the study sample.

To explore if the two groups were homogeneous at baseline, Student's *t*-test was used to compare both treatments (diltiazem and nifedipine) on the arterial blood pressure variables. In addition, pregnancy and maternity characteristics and liver and kidney function variables were analyzed using this test. Qualitative variables were compared using Fisher's exact test. All analyses employed IBM Statistics SPSS 21, while line graphs of two sets of group data were done in Sigma Plot. Statistical significance was reached at *p* < 0.05.

Comparisons between groups on the repeated measurements were made using repeated measures ANOVA [29]. Analyses included fixed effects for time (baseline and 6, 12, 18, 24, 30, 36, 42, and 48 hours), treatments (diltiazem and nifedipine), and treatments×time interactions. If interaction was statistically significant, post hoc analysis using the Sidak correction method for mean differences was performed to determine whether the changes in levels of arterial blood pressure between the longitudinal assessments were statistically significant between diltiazem and nifedipine treatments. When necessary, a Greenhouse–Geisser correction was applied to correct for nonsphericity.

Additionally, we fitted a binomial negative model to estimate the average number of hypotension episodes on days of hospitalization and incidence rate ratio. After fitting the model, residual deviance was used to perform a chi-square goodness of fit test for the overall model [30, 31].

## 3. Results

### 3.1. Patient Enrollment and Baseline Characteristics

**Figure 1:**
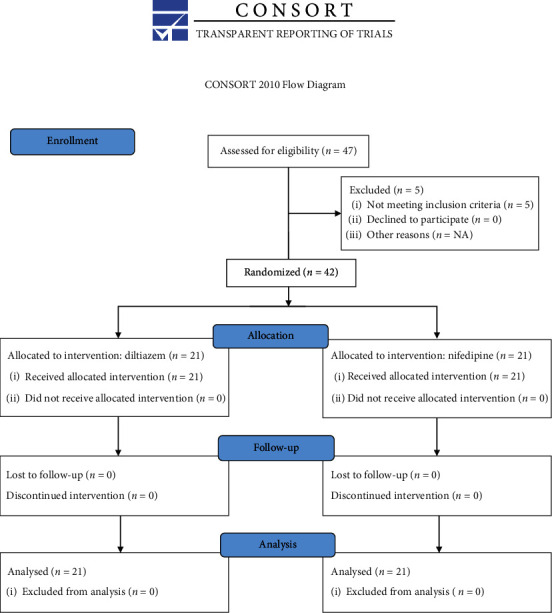
CONSORT chart for a trial of diltiazem and nifedipine for the control of blood pressure in puerperal patients with severe preeclampsia.

The trial flow diagram of patient enrollment is depicted in Figure 1. Among 47 hospitalized patients screened, 5 patients were not eligible because they did not meet inclusion criteria. A total of 42 puerperal patients with severe preeclampsia were finally randomized and completed the study.

Baselines characteristics of both groups were homogeneous (Table 1) with respect to maternal age, gestational age, childbirth delivery (vaginal passage or caesarean section), pregnancy (primiparous or multiparous), blood pressure (systolic and diastolic), mean arterial pressure, heart rate, and liver and kidney functions (AST, ATL, and serum creatinine). Also, baselines values of serum creatinine and transaminases were in normal range in all patients (Table 1).

### 3.2. Treatment Effect on Blood Pressure Parameters

Descriptive data of outcome measures by treatments and time point for blood pressure variables analyses are presented in Tables 2 and 3 and displays repeated measures ANOVA analyses for mean arterial pressure, blood pressure (systolic and diastolic), and heart rate. The analyses revealed significant effects for both principal effects (time and treatment) and the interaction effect (treatment×time), indicating actual effectiveness of the intervention on blood pressure variables.

**Figure 2:**
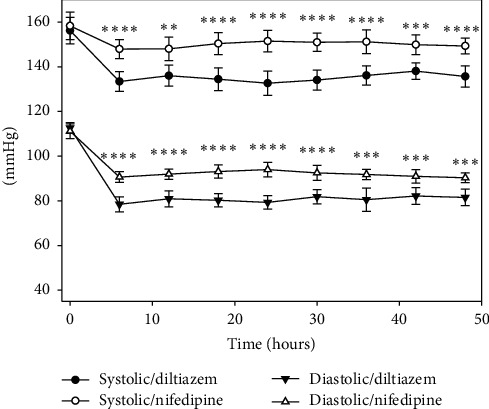
Systolic and diastolic blood pressure during 48 hours of observation, showing that both drugs reduced blood pressure. The decrease was greater with diltiazem. ^*∗*^*p* < 0.05, ^*∗∗*^*p* < 0.01, ^*∗∗∗*^*p* < 0.001, and ^*∗∗∗∗*^*p* < 0.001. Values expressed as the means ± 2SEM were evaluated by repeated measures ANOVA.

**Figure 3:**
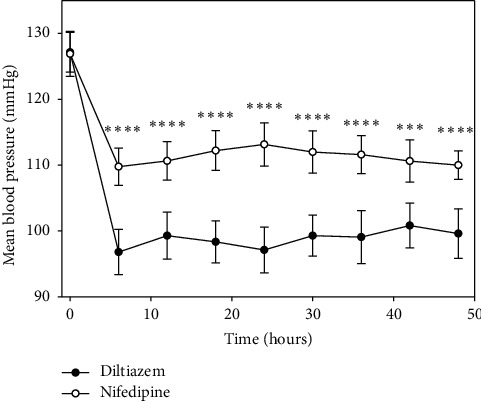
Mean blood pressure based on measurements of blood pressure with data sampled at 6-hour intervals for 48 hours, showing that both drugs lowered blood pressure. However, the reduction was greater with diltiazem. ^*∗*^*p* < 0.05, ^*∗∗*^*p* < 0.01, ^*∗∗∗*^*p* < 0.001, and ^*∗∗∗∗*^*p* < 0.001. Values expressed as the means ± 2SEM were evaluated by repeated measures ANOVA.

Analysis of primary outcome measures (systolic and diastolic blood pressure) showed that, in both study arms (diltiazem and nifedipine), there was no difference in baseline systolic blood pressure (SBP) (156.2 (13.6) vs. 158.3 (14.0), *p*=0.63) and baseline diastolic blood pressure (DBP) (112.6 (5.5) vs. 111.2 (7.6), *p*=0.49). At six hours, the difference of SBP (133.4 (10.2) vs. 147.9 (9.7)) and DBP (78.5 (7.7) vs. 90.6 (5.5)) was statistically significant between groups (*p* < 0.001 and *p* < 0.001, respectively). The trends of differences were evident in the following evaluations of both systolic pressure and diastolic pressure between diltiazem and nifedipine treatments (Figure 2 and Table 2). Similarly, basal mean arterial pressure (MAP) was not statistically significant between groups (*p*=0.62), and from six until 48 hours, the average of MAP was statistically significant between treatments (Figure 3).

### 3.3. Treatment Effect on Heart Rate

**Figure 4:**
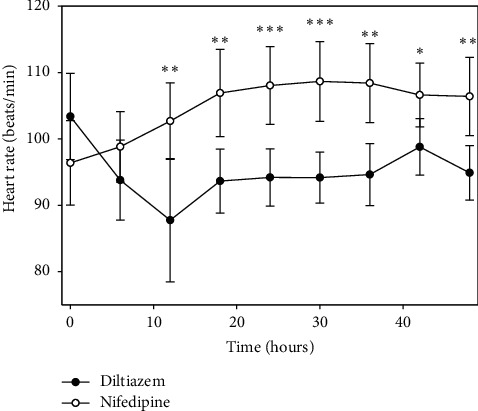
Average of heart rate during 48 hours of observation, showing distinct patterns for each drug with an increase and a decrease for patients with nifedipine and diltiazem, respectively. ^*∗*^*p* < 0.05, ^*∗∗*^*p* < 0.01, and ^*∗∗∗*^*p* < 0.001. Values expressed as the means ± 2SEM were evaluated by repeated measures ANOVA.

Recordings of the average heart rate every 6 hours for 48 hours are shown in Table 2 for patients of each group. The average of the basal heart rate was 103.4 (14.9) vs. 96.4 (14.6) (*p*=0.13) and at hour 6 was 93.8 (13.8) vs. 98.8 (12.2), (*p*=0.22); therefore, the difference was not statistically significant between the diltiazem and nifedipine groups. At all other times of heart rate recordings, the difference between groups was statistically significant (Figure 4).

### 3.4. Adverse Events

Analysis of secondary outcome measures was performed to compare collateral effects and the number of hypotension and hypertension episodes. The number of hypotension and hypertension episodes was statistically significant between groups (*p* < 0.001 and *p*=0.01, respectively). Whereas only 3 (14.3%) patients suffered hypotension episodes in the diltiazem group, and 15 (71.4%) patients suffered episodes of the same condition in the nifedipine group. On one hand, there were no hypertension episodes in the diltiazem group, which contrasts with 7 (33.3%) patients who suffered episodes of this condition in the nifedipine group.

We used a negative binomial (NB) regression model to estimate the average predicted count of hypotension episodes between treatments, which resulted in 0.238 and 2.095 in the diltiazem and nifedipine groups, respectively. Regression coefficients and standard error of the model are presented in Table 4. The NB model indicates that the incident rate ratio (IRR) of treatments (intervention with respect to control) is 0.114 (95% CI, 0.036–0.296).

### 3.5. Days in Intensive Care

Finally, patients in the diltiazem group spent an average of 2.47 (0.60) days in intensive care, while patients in the nifedipine group spent an average of 4.57 (1.36) days (*p* < 0.001). Thus, the total number of days in intensive care is lower in the diltiazem group, and these data could be used to estimate potential savings in this emergency therapy.

## 4. Discussion

Preeclampsia is a worldwide health problem with high prevalence in developing countries, including Mexico [2]. This disorder can lead to serious complications, including possible infant risk and death of the mother [32]. Proposed drugs for treatment are the L-type calcium channel antagonists [25, 33], such as nifedipine, which produces peripheral vasodilation [34]. In the present study, diltiazem was tested and compared to nifedipine during 48 hours of observation. Puerperal patients with severe preeclampsia were treated upon arrival at the intensive care unit of “Hospital de la Mujer” in Mexico City. Blood pressure monitoring was continuous, but data were recorded every 6 hours, showing a continuous decrease for both treatment groups. Within the 48-hour observation period, patients treated with nifedipine had a higher average decrease in blood pressure compared to the other group, but these values were in the upper range of the values reported in the literature.

According to Sibai [4, 34–37] and Brown [37, 38], the goal of disease management is to decrease blood pressure and avoid hypotension events. Furthermore, mean blood pressure should be diminished between fifteen and twenty-five percent until reaching a systolic pressure of 140 mmHg and a diastolic pressure of 90 mmHg. The objective of gradual reduction of blood pressure within this range is to avoid adverse events [19, 39, 40]. In the present study, the treatment that fulfilled this objective was diltiazem because within 48 hours no patient presented hypertension episodes (compared to 7 patients treated with nifedipine), and fewer subjects had hypotension episodes (3 versus 15 with nifedipine). For the oral nifedipine group, two previous trials [41, 42] reported hypotension episodes of 1/25 and 0/60, respectively. In our study, maternal hypotension was higher compared to previous trials.

A large number of nifedipine-related adverse episodes can be explained by its calcium channel blocking activity, which is 1000 times more potent than diltiazem [43]. The main pharmacological characteristic of nifedipine is nonselective vasodilation of arterial beds, affecting venous return mechanism and manifesting itself with clinical sign of hypotension. This rapid fall in blood pressure activates brainstem vasomotor centers, activating the sympathetic nervous system via efferent connections, hence producing peripheral vasoconstriction and an increase in heart rate and output [43, 44]. In this study, we can observe that diltiazem meets with international standards for the treatment of blood pressure in women with preeclampsia, decreasing systolic and diastolic pressure homogeneously. The incident rate of hypotension episodes for the diltiazem group is 0.036–0.296 times the incident rate for the control group.

Patients treated with diltiazem spent an average of 2.1 (95% CI, 1.4344, 2.7656) less days in intensive care. Hence, it can be inferred that by decreasing the length of stay in the intensive care unit (one of the most expensive services at the hospital level), costs would be favorably affected. However, a pharmacoeconomic analysis is necessary for evaluating this consideration.

Our study has some limitations. Providers were not blinded to treatment allocation. Also, we did not collect information on blood pressure parameters after discharge from the ICU. It would also have been interesting in conducting a longer observation of patients.

## 5. Conclusions

Administration of diltiazem (60 mg) was shown to be effective for controlling blood pressure in early puerperium patients with severe preeclampsia, leading to a more uniform control of arterial hypertension compared to nifedipine. Reduction in blood pressure was significantly greater in the diltiazem group than that in the nifedipine group during the 48-hour observation period. Finally, the improved blood pressure control produced with diltiazem was accompanied by less adverse episodes and less time in the intensive care unit.

## Figures and Tables

**Table 1:** Clinical and biochemical features of the treatments at baseline.

Variable	Diltiazem (*n* = 21)	Nifedipine (*n* = 21)	*p* value
Pregnancy and maternity characteristics			
Maternal age (years)	21.6 (6.8)	23.2 (6.2)	0.43
Gestational age (weeks)	36.4 (3.5)	36.4 (2.1)	0.99
Childbirth (caesarean section)	20/21 (95.2%)	21/21 (100%)	1.00
Pregnancy (primiparous)	16 (76.19%)	13 (61.90%)	0.51
Liver and kidney functions			
AST (U/L)	29.90 (10.36)	29.38 (7.34)	0.85
ALT (U/L)	20.90 (11.89)	30.47 (46.33)	0.36
Serum creatinine (mg/dL)	0.866 (0.229)	0.843 (0.159)	0.70
Baseline levels of arterial blood pressure variables			
Systolic blood pressure (mmHg)	156.2 (13.6)	158.3 (14.0)	0.63
Diastolic blood pressure (mmHg)	112.6 (5.5)	111.2 (7.6)	0.49
Mean blood pressure (mmHg)	127.1 (6.9)	126.9 (7.8)	0.91
Heart rate (beats/min)	103.4 (14.9)	96.4 (14.6)	0.13

AST: aspartate amino transferase; ALT: alanine amino transferase. Significant *p* values are bolded.

**Table 2:** Descriptive statistics of arterial blood pressure variables by treatments and time point.

Variable	Treatment	Basal time mean (SD)	6 h mean (SD)	12 h mean (SD)	18 h mean (SD)	24 h mean (SD)	30 h mean (SD)	36 h mean (SD)	42 h mean (SD)	48 h mean (SD)
Systolic blood pressure (mmHg)	Diltiazem	156.2 (13.6)	133.4 (10.2)	136.0 (10.8)	134.4 (11.6)	132.6 (12.4)	134.0 (10.3)	136.1 (9.7)	138.0 (8.4)	135.7 (10.9)
	Nifedipine	158.3 (14.0)	147.9 (9.7)	148.0 (12.0)	150.37 (11.2)	151.4 (10.9)	150.95 (9.4)	151.1 (12.2)	149.8 (10.2)	149.3 (8.2)
Diastolic blood pressure (mmHg)	Diltiazem	112.6 (5.5)	78.5 (7.7)	80.9 (8.3)	80.3 (6.8)	79.3 (6.9)	81.9 (7.2)	80.5 (12.0)	82.2 (8.6)	81.5 (8.5)
	Nifedipine	111.2 (7.6)	90.6 (5.5)	91.9 (5.3)	93.1 (6.8)	93.9 (7.5)	92.5 (7.7)	91.8 (5.4)	91.0 (6.9)	90.3 (5.1)
Mean blood pressure (mmHg)	Diltiazem	127.1 (6.9)	96.8 (7.9)	99.3 (8.2)	98.3 (7.3)	97.1 (8.0)	99.3 (7.1)	99.0 (9.2)	100.8 (7.8)	99.6 (8.6)
	Nifedipine	126.9 (7.8)	109.7 (6.5)	110.6 (6.7)	112.2 (6.9)	113.1 (7.6)	111.9 (7.4)	111.6 (6.6)	110.6 (7.4)	109.9 (5.0)
Heart rate (beats/min)	Diltiazem	103.4 (14.9)	93.8 (13.8)	87.7 (21.3)	93.6 (11.1)	94.2 (9.9)	94.2 (8.8)	94.6 (10.7)	98.8 (9.8)	94.9 (9.4)
	Nifedipine	96.4 (14.6)	98.8 (12.2)	102.7 (13.2)	106.9 (15.1)	108.0 (13.4)	108.6 (13.8)	108.4 (13.7)	106.6 (11.0)	106.4 (13.5)

**Table 3:** Results of repeated measures ANOVA for arterial blood pressure variables.

	Time			Treatment			Interaction		
	*F*	df	*p* value	*F*	df	*p* value	*F*	df	*p* value
Systolic blood pressure^*∗*^	15.955	5.250	<0.001	30.590	1	<0.001	3.651	5.250	**0.001**
Diastolic blood pressure^*∗*^	84.452	5.863	<0.001	47.569	1	<0.001	6.143	5.863	<**0.001**
Mean blood pressure^*∗*^	64.780	5.259	<0.001	53.325	1	<0.001	6.388	5.259	<**0.001**
Heart rate^*∗*^	2.639	4.352	0.03	12.037	1	0.001	5.351	4.352	<**0.001**

^*∗*^With a Greenhouse–Geisser correction. Significant *p* values are bolded.

**Table 4:** Effect of treatments on the number of episodes of hypotension.

	Variable	B	SE	95% IC		*p* value
				LL	LU	
Model^*∗*^	Intercept	0.740	0.224	0.301	1.184	**0.001**
	Treatments (diltiazem)	−2.175	0.527	−3.320	−1.216	<**0.001**

B: regression coefficient; SE: standard error; 95% CI: 95% confidence interval; LL: lower limit; LU: upper limit. ^*∗*^Dispersion parameter, 1.7228. Significant *p* values are bolded.

## Data Availability

The data used to support the findings of this study are available from the corresponding author upon request.
